# Correlation of clinical and radiological findings in patients with spinal trauma at Inkosi Albert Luthuli Central Hospital

**DOI:** 10.4102/sajr.v29i1.3248

**Published:** 2025-12-20

**Authors:** Lufuno J. Badzhi, Timothy C. Hardcastle, Pumersha Naidoo

**Affiliations:** 1 Department of Radiology, School of Medicine, University of KwaZulu-Natal, Durban, South Africa; 2 Department of Radiology, Victoria Mxenge Hospital, KwaZulu-Natal Department of Health, Durban, South Africa; 3 Department of Surgical Sciences, School of Medicine, University of KwaZulu-Natal, Durban, South Africa; 4 Trauma and Burns Unit, Inkosi Albert Luthuli Central Hospital, KwaZulu-Natal Department of Health, Durban, South Africa; 5 Department of Health, School of Medicine, University of KwaZulu-Natal, Durban, South Africa

**Keywords:** traumatic spinal cord injury, cervical spine injury, motor level, sensory level, MRI, CT scan, Glascow Comma Scale

## Abstract

**Background:**

Assessment of spinal trauma entails a full neurological examination and radiological assessment to determine the level of spinal cord injury.

**Objectives:**

This study aimed to determine if further imaging is always required, whether the clinical picture correlates with imaging results and to compare clinical and radiological prediction accuracy.

**Method:**

This retrospective chart review compared and correlated clinical findings with radiological findings in patients with spinal trauma at Inkosi Albert Luthuli Central Hospital over a period of 6 years. Demographics and sensitivity and specificity of clinical to imaging correlation with positive predictive ratios were assessed.

**Results:**

A total of 290 patients admitted with spinal injury, who received CT and/or MRI, were evaluated. Cervical-spine injuries were common. For predicting abnormal CT findings, the sensitivity of motor and sensory findings was 69.2% with a specificity of 85.4%. The positive predictive value (PPV) of motor and sensory findings was 96.2%. The negative predictive value (NPV) of motor and sensory findings was 34.0%. On MRI, sensitivity for motor and sensory findings was 85.1% for correctly predicting abnormal MRI findings, while the specificity was 52.8%. The PPV of motor and sensory findings was 82.5% with a NPV of 57.6%.

**Conclusion:**

In this trauma population, correlation of clinical findings with abnormal CT findings was 84.4% and for MRI findings was 72.3%, indicating that clinical findings alone may not be sufficient to rule out the need for imaging; false negatives could lead to missed or incorrect level of injury diagnoses.

**Contribution:**

This study adds to the proof that while clinical findings are reasonably accurate for the determination of neurological spinal cord injury level, both CT and MRI add additional information, making these tests invaluable.

## Introduction

There has been a marked increase in the incidence of spinal injury in spinal units in parts of Africa and the sub-Saharan region over the past years. South Africa has experienced a rise in the prevalence of trauma with associated spinal cord injuries. The admission rate in the spinal unit continues to rise, often exceeding the number of beds hospitals can provide. Most of these patients suffer cervical spine injuries, with thoracic and lumbar injuries being less common. The peak incidence age is between 21 and 30 years, followed by 30–40 years. There is a high male-to-female ratio. The prevalent mechanisms of injury include vehicle-related, with drunk driving accounting for almost half of the percentage, followed by pedestrian crashes, assault, falls and gunshots in descending order.^1,2,3^

A full neurological examination involves motor and sensory examination. Evaluation of the different dermatomes, myotomes and rectal examination completes the examination as detailed by the International Standards for Neurological Classification of Spinal Cord Injury (ISNCSCI). In the past, Frankel’s assessment classification was used to score patients clinically to predict the level, severity and outcome associated with the spinal cord injury. However, since 1992, Frankel’s guidelines were modified to the American Spinal Injury Association (ASIA) because of the observed inconsistency in accuracy in patients with complete spinal injuries. The use of ISNCSCI in association with the ASIA impairment scale clinically predicts the severity and the outcome of the spinal cord injury, complemented by imaging.^4,5^

The Canadian C-spine rule (CCR) and the National Emergency X-Radiography Utilization Study (NEXUS) criteria are the two rules that are used to assist emergency physicians in assessing the need for cervical spine imaging. The use of each guideline or the combination of both has shown greater efficiency in predicting spinal cord injuries. However, the use of these algorithms does not supersede the need for imaging in patients with a high index of suspicion.^6,7,8^ In the cases of the patients included in this study, the NEXUS and CCR do not apply, as all the patients either had neck pain or had neurological signs.

All patients who are clinically suspected to have spinal trauma will be imaged by X-rays complemented by CT and selectively with MRI or vascular imaging per protocol, depending on the anatomical location. The vertebral column provides the framework and stabilises the axial skeleton. It also houses and protects neural structures, such as the spinal cord, nerve roots, and cauda equina. Promptly recognising spinal injuries clinically will guide treatment, which will guide surgical decision-making. Familiarity with radiography, CT and MRI in evaluating spinal trauma is necessary. In some clinical settings, all three methods are useful in determining management and surgical planning.^9^

The imaging technique of choice is MRI, particularly if CT demonstrates no pathology in a patient with neurological fallout. An MRI is ideal for the evaluation of spinal cord injury and para-spinal soft tissue injuries, while it is less accurate for bone pathology, for which a CT scan is very reliable and allows for 3D reconstructions. However, the use of MRI in all patients with spinal trauma is not practical as it is not easily available and is costly. Therefore, MRI is performed selectively based on the clinical, radiographic and CT findings.^10^

This study aimed to assess the ability of the trauma teams at Inkosi Albert Luthuli Central Hospital to clinically examine and approximate the level of the neurological status in patients with spinal injuries with neurological deficits by correlating their findings with the injuries identified during the radiological assessment. Objectives included firstly to determine whether imaging is always warranted based on clinical findings to improve patient care and generate protocols. Secondly, to evaluate whether the clinical findings are reliably able to approximate the level of neurological damage when correlated with imaging modalities. Thirdly, to compare the findings of the physical examination performed in the emergency trauma unit with the accuracy thereof when compared to the radiological assessment. Finally, to determine the confounding factors affecting the clinical examination findings necessitating radiological assessment.

## Research methods and design

A retrospective observational descriptive study was conducted. All consecutive, eligible patients seen in the Trauma Unit between January 2018 and December 2024 (6 years) with traumatic spinal injuries and neurological deficit, who received imaging with CT and/or MRI, were included.The radiological data (X-rays, CT and MRI) were obtained from the stored hospital database picture archiving and communications system (PACS), for the study period. The images and the reports were reviewed.

The CT scans were undertaken with a Flash Somatom 256 dual source scanner (Siemens, Germany, Munich), with appropriate reconstructions of the spine in the axial, coronal and sagittal planes, using a 3mm x 3mm slice, window level inner ear, Kenel 70 sharp with 1 mm × 0.8 mm – on SyngoVIA and with 3D reconstructions of all fractures.

The MRI sequences performed on a 1.5 Tesla included whole spine sagittal T2-weighted (T2W) and short tau inversion recovery (STIR). At the level of injury, additional T2W (sagittal and axial), T2W axial trace haemorrhage sequence and T1W (sagittal and axial) sequences were done as routine. Time of Flight MRA neck vessels was performed for suspected vascular injury. If injury was noted over the C1–C2 vertebral region, then T2W coronal imaging was performed. For C-Spine trauma with brachial plexus injury, routine C-spine T2 sagittal, T1W sagittal, T2W trace and T2W sagittal 3D constructive interference in steady state (CISS) were acquired. Dedicated obliques for brachial plexus injury views were not performed.

Clinical data were obtained from patient clinical charts, covered by the Biomedical Research Ethics Committee (BREC)-approved Trauma Registry. Patient confidentiality was maintained and no patient contact was required. Data were de-identified at the source.

### Data management

The Microsoft Excel (Microsoft Corp, Redmond, WA, United States) database was used to consolidate data. All data were electronically accrued and access was password-protected. As per the University of KwaZulu-Natal regulations and BREC rules, data will be retained for 5 years.

### Statistical analysis

Descriptive statistics were used to summarise patient demographic characteristics, clinical findings and CT/MRI findings. Data were stratified by sensory, motor, CT and MRI findings to facilitate comparisons across these groups and assess potential differences in demographic and injury-related characteristics. Non-normally distributed quantitative data were presented as medians with interquartile ranges (IQRs) and compared using the Mann–Whitney U test. Categorical data were expressed as frequencies with percentages and compared using the Chi-square test. Data were also presented graphically where appropriate.

The diagnostic performance of clinical findings in predicting abnormal radiological findings was evaluated using standard diagnostic measures, including sensitivity, specificity, positive predictive value (PPV) and negative predictive value (NPV). Sensitivity represented the proportion of actual positives correctly identified by clinical findings (i.e. motor or sensory), while specificity indicated the proportion of actual negatives correctly identified. Positive predictive value reflected the probability that patients with positive clinical findings truly had abnormal radiological findings (i.e. on CT or MRI), whereas NPV indicated the probability that patients with negative clinical findings truly had normal radiological findings.

To evaluate agreement between clinical and radiological findings, a concordance and discordance analysis was performed using a 2 × 2 contingency table, with comparisons made using McNemar’s test. Agreement between clinical and radiological findings was further assessed using Cohen’s kappa statistic, with interpretation based on Cohen’s guidelines: ≤ 0.20 (slight agreement), 0.21–0.40 (fair agreement), 0.41–0.60 (moderate agreement), 0.61–0.80 (substantial agreement), and 0.81–1.00 (almost perfect agreement).

Cohen’s kappa was chosen because it adjusts for the level of agreement expected to occur by chance. Unlike simple percentage agreement, it offers a more robust and interpretable measure of concordance between two raters or diagnostic methods, especially in situations where marginal distributions are imbalanced. Confidence intervals (CIs) are listed in the updated tables.

A two-tailed *p*-value at 5% was considered to indicate statistical significance for all analyses. All statistical analyses were performed using Stata software version 18 (StataCorp LLC, College Station, TX, United States).

### Ethical considerations

Ethical clearance to conduct this study was obtained from the University of KwaZulu-Natal BREC as a pre-approved sub-study of the Trauma and Burns Class Approval for retrospective research at Inkosi Albert Luthuli Central Hospital. (reference number: BCA207/09).

## Results

A total of 808 patient charts were reviewed and 290 patients met the inclusion criteria for spinal injury. Of these, 242 (83.7%) were male patients. The median age was 33 years with an IQR of 26 years – 43 years. Cervical injuries were observed clinically in 221 patients (76.5%), while 40 (13.8%) had thoracic spine injuries, lumbo-sacral or multilevel injuries amounted to almost 10% (Table 1). This pattern was statistically significant with *p*-values of 0.001 for motor signs and 0.006 for sensory deficits.

**TABLE 1:** Demographic and injury characteristics by sensory and motor injury findings (*N* = 289).

Variables	Positive (*n* = 156)		Negative (*n* = 106)		Not performed (*n* = 27)		Total		*p*-value
	*n*	%	*n*	%	*n*	%	*n*	%	
**Anatomical location**	-	-	-	-	-	-	-	-	0.006*
C-spine	110	70.5	88	83.0	23	85.2	221	76.5	-
T-spine	32	20.5	5	4.7	3	11.1	40	13.8	-
L-spine	6	3.8	1	0.9	1	3.7	8	2.8	-
C-spine + T-spine	6	3.8	5	4.7	0	0.0	11	3.8	-
T-spine + L-spine	1	0.6	1	0.9	0	0.0	2	0.7	-
C-spine + T-spine + L-spine	0	0.0	6	5.7	0	0.0	6	2.1	-
C-spine + L-spine	1	0.6	0	0.0	0	0.0	1	0.3	-
**Glasgow Coma Score (GCS) categories**	-	-	-	-	-	-	-	-	0.014*
Severe injury (GCS 1–8)	24	15.4	22	20.8	12	44.4	58	20.1	-
Moderate injury (GCS 9–12)	18	11.5	13	12.3	3	11.1	34	11.8	-
Mild injury (GCS 13–15)	114	73.1	71	67.0	12	44.4	197	68.2	-
**Mechanism of injury**	-	-	-	-	-	-	-	-	0.230
Motor Vehicle Collision (MVC)	63	40.6	44	41.5	14	51.9	121	42.0	-
Pedestrian Vehicle Crash (PVC)	24	15.5	28	26.4	5	18.5	57	19.8	-
Gunshot	28	18.1	6	5.7	2	7.4	36	12.5	-
Fall from height	30	19.4	20	18.9	3	11.1	53	18.4	-
Assault	6	3.9	4	3.8	2	7.4	12	4.2	-
Stab	3	1.9	3	2.8	1	3.7	7	2.4	-
Unknown	1	0.6	1	0.9	0	0.0	2	0.7	-

* , Statistical significance.

There were statistically significant differences in Glasgow Coma Scores (GCS), with the majority of patients falling within the 13–15 range. Potential confounding injuries were present in the polytrauma patients, numbering 210, of whom more than 70% had concurrent spinal and brain injuries, 109 (37.5%) chest, 83 (28.6%) abdominal and 108 (37.2%) limb injuries. According to the distribution of mechanisms of injury, the vast majority (61.8%) were victims of either a motor vehicle collision (MVC) or pedestrian vehicle crash (PVC), while gunshot and falls from height were the next most common mechanisms (Table 1).

Logistic regression was used to identify factors associated with positive or abnormal clinical findings. The multivariable model adjusted for potential confounders such as age, sex, mechanism of injury and GCS category, with results presented as adjusted odds ratios (aOR) at 95% CI (Table 2). There were no significant correlations. Table 3 and Table 4 detail the clinical findings and related diagnostic imaging findings.

**TABLE 2:** Regression analysis: Factors associated with positive and negative motor and sensory findings.

Characteristics	Motor findings						Sensory findings					
	Univariable			Multivariable			Univariable			Multivariable		
	OR	95% CI	*p*-value	aOR	95% CI	*p*-value	OR	95% CI	*p*-value	aOR	95% CI	*p*-value
Age	0.99	0.84–1.17	0.910	0.98	0.81–1.17	0.802	0.95	0.80–1.13	0.600	0.94	0.79–1.14	0.551
**Sex**												
Male	1.50	0.77–2.92	0.231	1.32	0.65–2.70	0.440	1.59	0.82–3.08	0.171	1.43	0.71–2.92	0.316
Female	Ref	Ref	Ref	Ref	Ref	Ref	Ref	Ref	Ref	Ref	Ref	Ref
**Glasgow Coma Score (GCS) categories**												
Severe injury (GCS 1–8)	0.57	0.30–1.01	0.088	0.76	0.37–1.55	0.450	0.67	0.35–1.30	0.244	0.93	0.45–1.90	0.843
Moderate injury (GCS 9–12)	0.79	0.36–1.70	0.542	1.06	0.46–2.47	0.888	0.86	0.40–1.87	0.707	1.18	0.51–2.73	0.707
Mild (GCS 13–15)	Ref	Ref	Ref	Ref	Ref	Ref	Ref	Ref	Ref	Ref	Ref	Ref
**Mechanism of injury**												
Motor Vehicle Collision (MVC)	1.55	0.09–25.42	0.760	1.49	0.09–26.0	0.783	1.43	0.09–23.51	0.802	1.55	0.09–27.10	0.761
Pedestrian Vehicle Crash (PVC)	0.86	0.05–14.45	0.915	0.85	0.05–14.93	0.909	0.86	0.05–14.45	0.915	0.90	0.05–15.89	0.943
Gunshot	4.67	0.25–85.55	0.299	4.59	0.23–91.41	0.319	4.67	0.25–85.55	0.299	5.21	0.26–103.95	0.280
Fall from height	1.63	0.09–27.65	0.735	1.60	0.09–29.29	0.750	1.50	0.09–25.39	0.779	1.69	0.09–30.92	0.722
Assault	1.50	0.07–31.57	0.794	1.33	0.06–29.56	0.856	1.50	0.07–31.57	0.794	1.44	0.06–1.90	0.818
Stab	1.00	0.04–24.55	1.000	0.97	0.04–24.92	0.983	1.00	0.04–24.55	1.000	1.07	0.04–27.67	0.967
Unknown	Ref	Ref	Ref	Ref	Ref	Ref	Ref	Ref	Ref	Ref	Ref	Ref

OR, odds ratio; aOR, adjusted odds ratio; CI, confidence interval; Ref, reference.

**TABLE 3:** Concordance between clinical (motor and sensory) and radiological findings (CT and MRI).

Variable	Negative		Positive		Total	
	*n*	%	*n*	%	*n*	%
**CT findings**						
**Motor findings†**						
Negative	35	13.4	6	2.3	41	15.7
Positive	68	26.0	153	58.4	221	84.4
**Total**	**103**	**39.3**	**159**	**60.7**	**262**	**100.0**
**Sensory findings‡**						
Negative	34	13.0	7	2.7	41	15.7
Positive	72	27.5	149	56.9	221	84.4
**Total**	**106**	**40.5**	**156**	**59.5**	**262**	**100.0**
**MRI findings**						
**Motor findings§**						
Negative	19	14.6	17	13.1	36	27.7
Positive	14	10.8	80	61.5	94	72.3
**Total**	**33**	**25.4**	**97**	**74.6**	**130**	**100.0**
**Sensory findings¶**						
Negative	19	14.2	17	13.1	36	27.7
Positive	18	13.9	76	58.5	94	72.3
**Total**	**37**	**28.5**	**93**	**71.5**	**130**	**100.0**

CT, computed tomography; MRI, magnetic resonance imaging.

† , Kappa and agreement: 0.34 (*p* < 0.001); 71.8%;

‡ , Kappa and agreement: 0.31 (*p* < 0.001); 69.9%;

§ , Kappa and agreement: 0.39 (*p* < 0.001); 76.2%;

¶ , Kappa and agreement: 0.33 (*p* < 0.001); 73.1%.

**TABLE 4:** Diagnostic performance of clinical findings in predicting abnormal CT and MRI findings.

Clinical findings	Sensitivity		Specificity		Positive predictive value		Negative predictive value	
	%	95% CI	%	95% CI	%	95% CI	%	95% CI
**CT†**								
Motor	69.2	62.7–75.2	85.4	70.8–94.4	96.2	92.0–98.6	34.0	24.9–44.0
Sensory	67.4	60.8–73.6	82.9	67.9–92.8	95.5	91.0–98.2	32.1	23.3–41.8
**MRI‡**								
Motor	85.1	76.3–91.6	52.8	35.5–69.6	82.5	73.4–89.4	57.6	39.2–74.5
Sensory	80.9	71.4–88.2	52.8	35.5–69.6	81.7	72.4–89.0	51.4	34.4–68.1

CI, confidence interval; CT, computed tomography; MRI, magnetic resonance imaging.

† , CT Prevalence: 84.4%, 95% CI 79.4–88.5;

‡ , MRI Prevalence: 72.3%, 95% CI 63.8–79.8.

## Discussion

This study showed a higher incidence of cervical spine injury at 76.5%, which was more than the documented figure in the literature of 50%. The frequent mechanism of injury was blunt force secondary to MVC and PVC, likely because of the study hospital being a referral centre for major trauma.^11^

During data collection, it was observed that spinal trauma usually occurred in conjunction with other organ injuries and was usually detected on polytrauma CT imaging as detailed in the results. Concurrent traumatic brain and C-spine injuries were frequent. This has been elaborated in a study that was previously performed in KwaZulu-Natal, which observed approximately 70% of concurrent injuries.^12^ Dedicated thoracic spine and lumbar spine CT was not performed on patients who had multi-organ injuries and received polytrauma CT. Bone reconstructions were performed and were adequate for interpretation as per the literature and the protocols currently in existence. This reduced time, storage space, and radiation exposure.^13^

There was a higher PPV of positive CT scan findings in imaged patients suspected to have spinal injuries clinically, suggesting a reasonable correlation with clinical suspicion but also showing that clinical signs can overestimate the injury level or position. A lower NPV suggests that negative motor and sensory findings are less reliable in predicting the absence of abnormal CT findings. There was a good level of reliability for positive test results, indicating that motor and sensory findings are reliable in predicting abnormal MRI outcomes. The NPV of motor and sensory findings indicates that when motor findings are moderately negative, the negative motor findings are not particularly reliable in excluding abnormal MRI results. MRI examinations with abnormal findings correlating with the clinical findings were observed; however, the clinical suspicion of injury was again higher than the identified injury rates; findings are detailed in Table 4.

Several issues were of particular concern: who should receive CT, MRI or radiographs, as some patients who were deemed to have abnormal neurology had normal imaging results and vice versa. Neurological examination entailed motor and sensory examination. Motor and sensory findings were always documented together and could not be used individually for clinical assessment. It was also observed that both clinical and radiological assessments complemented each other for good outcomes. Another issue of concern was the different intervals in obtaining MRI scans. This was attributed to the referral systems based on patient factors, administrative factors and clinical factors. Patients with abnormal neurology clinically and found to have no abnormalities on early MRI are expected to have a repeat MRI after 72 h. However, most patients had delayed presentations to the MRI department because of the aforementioned factors.

**FIGURE 1:**
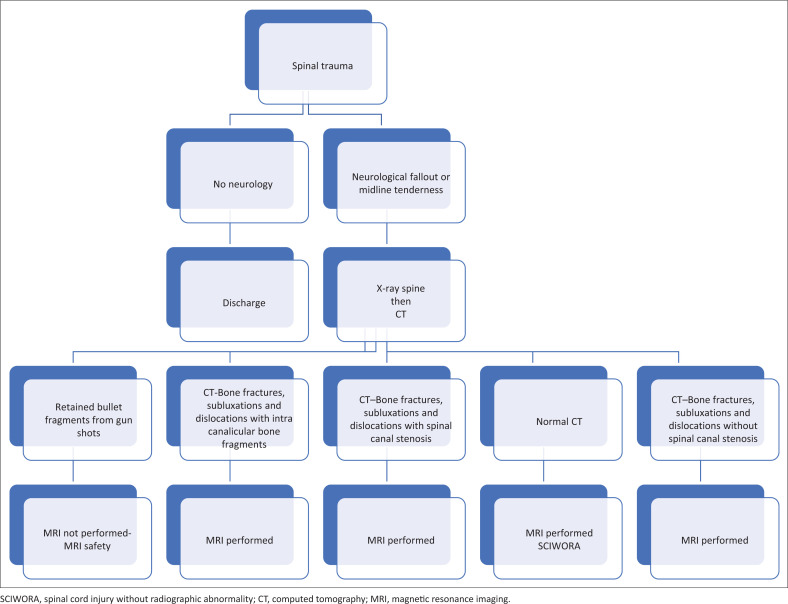
Imaging decision tree.

The concept of SCIWORA (Spinal Cord Injury Without Radiographic Abnormality) describes traumatic spinal injury presenting with neurological fallout clinically, with no radiological evidence on X-rays and CT which is usually noted in children.^14,15,16^ MRI was performed on a case-by-case basis when there was a high index of clinical suspicion, as demonstrated by the algorithm in Figure 1.

The CT scan was superior in demonstrating bone fractures, subluxations and dislocations. Documentation on the integrity of the spinal canal was essential. An MRI was superior in demonstrating posterior ligamentous injuries (PLC) and anterior ligamentous injury (ALC), spinal cord oedema and haemorrhages. Prevertebral haematoma, MRI T2W abnormal signal intensity and PLC injury had a high sensitivity for diagnosing cervical disc rupture. This was also detailed in a study performed in China, which suggested that prevertebral haematoma, spinal cord injury and PLC injury can be used as indicators to suggest disc rupture. The level of spinal cord injury can be used to locate the segment of the ruptured disc.^17^

**FIGURE 2:**
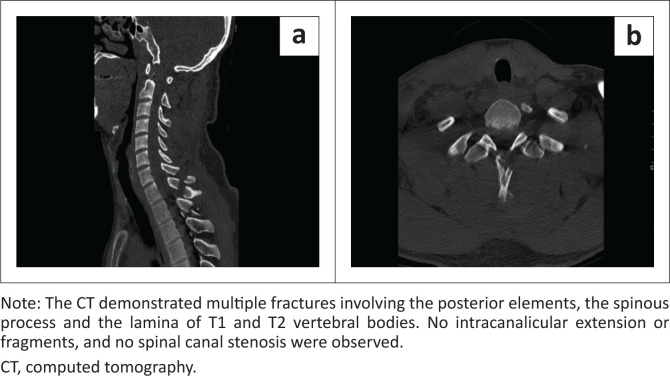
CT C-spine (a) sagittal reconstruction and (b) axial view of a patient who presented with neurological fall out, quadriplegia.

**FIGURE 3:**
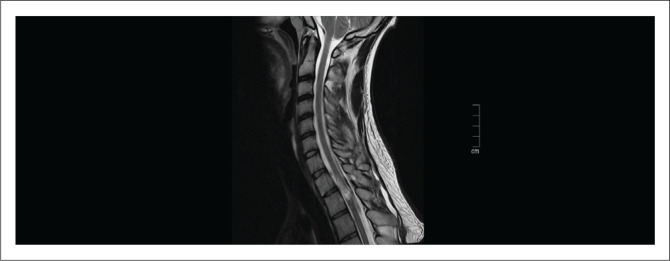
T2W sagittal sequence performed in the same patient after 3 days, showing spinal cord oedema in the upper thoracic spine demonstrating T2W signal hyperintensity.

Several studies have been performed to explain the superiority of MRI over CT when evaluating soft tissues of the spine in trauma patients and the benefit of synergistic use of both modalities, depending on the symptomatology.^18,19^ The use of CT alone was inadequate to completely exclude spinal cord injuries in some of the symptomatic patients. An MRI was able to detect subtle or silent soft tissue injuries, like ligamentous, disc and paraspinal soft tissues, with spinal cord oedema or haemorrhage, even in patients without spinal canal compromise (Figure 2 and Figure 3).^20,21^

Management of patients with spinal trauma begins at the incident scene with spinal motion restriction, traditionally with the use of the C-spine collar, although this has been recently questioned. Appropriate transportation into the emergency room and resuscitation according to the Advanced Trauma Life Support guidelines follows.^22^ In cases of suspected spinal cord injury, after a full neurological examination during the ‘secondary survey’, the patient is imaged and the results provide guidance for a conservative or a surgical approach to the management, depending on the findings.^23,24^

Advancements in spinal cord decompression with surgical instruments has significantly improved the surgical management of unstable spinal fractures. Regardless of the severity of injury, literature now favours early spinal cord surgical decompression and stabilisation within 24 h of trauma to the spine.^25^ Patients who had early surgical intervention for post-compressive myelopathy seen on MRI showed improvement after surgery.^26^

Limitations of the study included variability in clinical examination, potentially being inconsistent because of the differences in the level of clinical experience. However, severity might have been reduced because of the utilisation of the standardised guidelines adopted by the institution. This study was performed at a single centre as a retrospective study and therefore suffers from potential bias. This is mitigated by using an electronic record system based on a template record. Furthermore, as it is observational, no association with causality can be drawn.

## Conclusion

Vehicle-related trauma remains the leading cause of spinal injury, with a predominance of cervical spine involvement. Clinical examination alone cannot reliably exclude injury. Findings indicate that clinical findings overestimate the injury level. Concurrent multiorgan (brain, abdomen, chest and limb) injuries are frequent. A comprehensive neurological examination, complemented by a thorough radiological examination, provides a proper guide for a good management plan, which includes surgical intervention, conservative management and multidisciplinary teams (physiotherapy, occupational therapist, psychologist and social workers).
